# Supplementary material for: Pyruvate Dehydrogenase Kinase 1 inhibition mediated oxidative phosphorylation enhancement in cartilage promotes osteoarthritis progression
Source: BMC Musculoskelet Disord. 2023 Jul 20;24:597. doi: 10.1186/s12891-023-06585-6 (PMC10357736; doi:10.1186/s12891-023-06585-6)
Supplement: Supplementary file 1 — Additional file 1. [file 12891_2023_6585_MOESM1_ESM.zip › BP.Gsea.1653623667859/GOBP_ENDOSOME_TRANSPORT_VIA_MULTIVESICULAR_BODY_SORTING_PATHWAY.html]

Details for gene set GOBP\_ENDOSOME\_TRANSPORT\_VIA\_MULTIVESICULAR\_BODY\_SORTING\_PATHWAY[GSEA]

|  || Dataset | 55457\_collapsed\_to\_symbols.cls.cls #OA\_versus\_normal.cls.cls #OA\_versus\_normal\_repos |
| Phenotype | cls.cls#OA\_versus\_normal\_repos |
| Upregulated in class | OA |
| GeneSet | GOBP\_ENDOSOME\_TRANSPORT\_VIA\_MULTIVESICULAR\_BODY\_SORTING\_PATHWAY |
| Enrichment Score (ES) | 0.53051233 |
| Normalized Enrichment Score (NES) | 1.7857016 |
| Nominal p-value | 0.009070295 |
| FDR q-value | 0.08700169 |
| FWER p-Value | 0.99 |
Table: GSEA Results Summary

  

Fig 1: Enrichment plot: GOBP\_ENDOSOME\_TRANSPORT\_VIA\_MULTIVESICULAR\_BODY\_SORTING\_PATHWAY      
 Profile of the Running ES Score & Positions of GeneSet Members on the Rank Ordered List

  

| SYMBOL | TITLE | RANK IN GENE LIST | RANK METRIC SCORE | RUNNING ES | CORE ENRICHMENT || 1 | CHMP2B | charged multivesicular body protein 2B [Source:HGNC Symbol;Acc:HGNC:24537] | 129 | 0.876 | 0.0987 | Yes |
| 2 | LEPROTL1 | leptin receptor overlapping transcript like 1 [Source:HGNC Symbol;Acc:HGNC:6555] | 434 | 0.691 | 0.1609 | Yes |
| 3 | CHMP4A | charged multivesicular body protein 4A [Source:HGNC Symbol;Acc:HGNC:20274] | 526 | 0.649 | 0.2344 | Yes |
| 4 | CHMP5 | charged multivesicular body protein 5 [Source:HGNC Symbol;Acc:HGNC:26942] | 556 | 0.638 | 0.3113 | Yes |
| 5 | TMEM50A | transmembrane protein 50A [Source:HGNC Symbol;Acc:HGNC:30590] | 576 | 0.631 | 0.3882 | Yes |
| 6 | TMEM50B | transmembrane protein 50B [Source:HGNC Symbol;Acc:HGNC:1280] | 883 | 0.547 | 0.4324 | Yes |
| 7 | CHMP2A | charged multivesicular body protein 2A [Source:HGNC Symbol;Acc:HGNC:30216] | 1269 | 0.464 | 0.4600 | Yes |
| 8 | VPS28 | VPS28 subunit of ESCRT-I [Source:HGNC Symbol;Acc:HGNC:18178] | 1522 | 0.426 | 0.4933 | Yes |
| 9 | CHMP6 | charged multivesicular body protein 6 [Source:HGNC Symbol;Acc:HGNC:25675] | 2140 | 0.345 | 0.4883 | Yes |
| 10 | CHMP3 | charged multivesicular body protein 3 [Source:HGNC Symbol;Acc:HGNC:29865] | 2733 | 0.277 | 0.4767 | Yes |
| 11 | VPS4B | vacuolar protein sorting 4 homolog B [Source:HGNC Symbol;Acc:HGNC:10895] | 2783 | 0.271 | 0.5066 | Yes |
| 12 | SNF8 | SNF8 subunit of ESCRT-II [Source:HGNC Symbol;Acc:HGNC:17028] | 3037 | 0.246 | 0.5175 | Yes |
| 13 | SORT1 | sortilin 1 [Source:HGNC Symbol;Acc:HGNC:11186] | 3481 | 0.204 | 0.5084 | Yes |
| 14 | VCP | valosin containing protein [Source:HGNC Symbol;Acc:HGNC:12666] | 3518 | 0.201 | 0.5305 | Yes |
| 15 | UBXN6 | UBX domain protein 6 [Source:HGNC Symbol;Acc:HGNC:14928] | 4401 | 0.121 | 0.4770 | No |
| 16 | LEPROT | leptin receptor overlapping transcript [Source:HGNC Symbol;Acc:HGNC:29477] | 5608 | 0.023 | 0.3863 | No |
| 17 | STAM | signal transducing adaptor molecule [Source:HGNC Symbol;Acc:HGNC:11357] | 6403 | -0.035 | 0.3290 | No |
| 18 | CHMP1B | charged multivesicular body protein 1B [Source:HGNC Symbol;Acc:HGNC:24287] | 7339 | -0.105 | 0.2695 | No |
| 19 | LYST | lysosomal trafficking regulator [Source:HGNC Symbol;Acc:HGNC:1968] | 8343 | -0.181 | 0.2141 | No |
| 20 | CHMP7 | charged multivesicular body protein 7 [Source:HGNC Symbol;Acc:HGNC:28439] | 9723 | -0.294 | 0.1435 | No |
| 21 | LAPTM4B | lysosomal protein transmembrane 4 beta [Source:HGNC Symbol;Acc:HGNC:13646] | 10218 | -0.343 | 0.1477 | No |
| 22 | CHMP1A | charged multivesicular body protein 1A [Source:HGNC Symbol;Acc:HGNC:8740] | 11401 | -0.487 | 0.1164 | No |
Table: GSEA details [plain text format]

  

Fig 2: GOBP\_ENDOSOME\_TRANSPORT\_VIA\_MULTIVESICULAR\_BODY\_SORTING\_PATHWAY      
 Blue-Pink O' Gram in the Space of the Analyzed GeneSet

  

Fig 3: GOBP\_ENDOSOME\_TRANSPORT\_VIA\_MULTIVESICULAR\_BODY\_SORTING\_PATHWAY: Random ES distribution      
 Gene set null distribution of ES for **GOBP\_ENDOSOME\_TRANSPORT\_VIA\_MULTIVESICULAR\_BODY\_SORTING\_PATHWAY**
